# Case Report: Vasculitis Triggered by SIRT in a Patient With Previously Untreated Cholangiocarcinoma

**DOI:** 10.3389/fonc.2021.755750

**Published:** 2021-12-16

**Authors:** Antonia Stamatiou, Jeremy Jankovic, Petr Szturz, Francois Fasquelle, Rafael Duran, Niklaus Schaefer, Antonella Diciolla, Antonia Digklia

**Affiliations:** ^1^ Department of Oncology, Lausanne University Hospital (CHUV), Lausanne, Switzerland; ^2^ University Institute of Pathology, Clinical Pathology Service, Lausanne University Hospital (CHUV), Lausanne, Switzerland; ^3^ Department of Diagnostic Radiology and Interventional Radiology, Lausanne University Hospital (CHUV), Lausanne, Switzerland; ^4^ Faculty of Biology and Medicine, University of Lausanne, Lausanne, Switzerland; ^5^ Department of Nuclear Medicine and Molecular Imaging, Lausanne University Hospital (CHUV), Lausanne, Switzerland

**Keywords:** vasculitis, radioembolization, case report, cholangiocarcinoma, SIRT, cancer

## Abstract

Arising from the biliary tract, cholangiocarcinoma is a rare and aggressive epithelial cancer. According to the primary site, it can be further classified into intrahepatic, perihilar and distal types. Due to the lack of symptoms early in the disease course, most patients are diagnosed at advanced stages. Being not candidates for curative surgical management, these patients are treated with palliative systemic chemotherapy, and their prognosis remains poor. Using radioisotopes like yttrium-90 -labeled microspheres (^90^Y), radioembolization represents a local approach to treat primary and secondary liver tumors. In the case of intrahepatic cholangiocarcinoma, radioembolization can be used as a primary treatment, as an adjunct to chemotherapy or after failing chemotherapy. An 88-year-old man underwent radioembolization for a previously untreated stage II intrahepatic cholangiocarcinoma. One week later, he presented to our clinic with a non-pruritic maculopapular rash of the lower extremities and abdomen, worsening fatigue and low-grade fever. Laboratory exams, including hepatitis screening, were within normal limits. Showing positive immunofluorescence staining for immunoglobulin M (IgM) and complement 3 (C3) in vessel walls without IgA involvement, the skin biopsy results were compatible with leukocytoclastic vasculitis. Apart from the anticancer intervention, there have been no recent medication changes which could explain this complication. Notably, we did not observe any side effects during or after the perfusion scan with technetium-99m macroaggregated albumin (MAA) performed prior to radioembolization. The symptoms resolved quickly after a short course of colchicine and did not reappear at cholangiocarcinoma progression. In the absence of other evident causes, we conclude that the onset of leukocytoclastic vasculitis in our patient was directly linked to the administration of yttrium-90 -labeled microspheres. Our report therefore demonstrates that this condition can be a rare but manageable complication of ^90^Y liver radioembolization.

## Introduction

Cholangiocarcinoma is a rare and aggressive epithelial cancer that arises from the biliary tract and is classified into intrahepatic, perihilar and distal cholangiocarcinoma according to its localization. Early intrahepatic cholangiocarcinoma is usually asymptomatic. Therefore, most patients are diagnosed at advanced stages: they are not candidates for curative treatment (surgery) and have a median overall survival (mOS) < 8 months without treatment (1). Systemic chemotherapy with cisplatin and gemcitabine is the standard of care for patients with advanced cholangiocarcinoma, leading to an OS’s increase. However, the prognosis remains poor, leading to alternative treatments research (2, 3).

Local therapies such as radiofrequency ablation, chemoembolization and radioembolization are frequently used in the treatment of localized liver cancers and cholangiocarcinoma (1). They represent a valid alternative to surgery, when the latter cannot be performed due to the extent of the liver infiltration or the performance status of the patient.

We present the case of an elderly patient with intrahepatic cholangiocarcinoma who suffered from an episode of leukocytoclastic vasculitis following treatment with Yttrium 90 (^90^Y) radioembolization.

## Case Report

An 88 year-old man presented to the outpatient oncology clinic in April 2020 with a 48 hours history of non-pruritic rash on both legs and abdomen, worsening fatigue and low-grade fever, one week after radioembolization for a previously untreated well differentiated stage II intrahepatic cholangiocarcinoma in segment IV which had been diagnosed 2 months earlier. Other comorbidities included hypertension treated with calcium-channel blocker and angiotensin-converting enzyme inhibitors, without recent changes and localized prostate cancer treated by radiotherapy 15 years ago and considered in remission since then. He had no known allergy.

**Figure 1:**
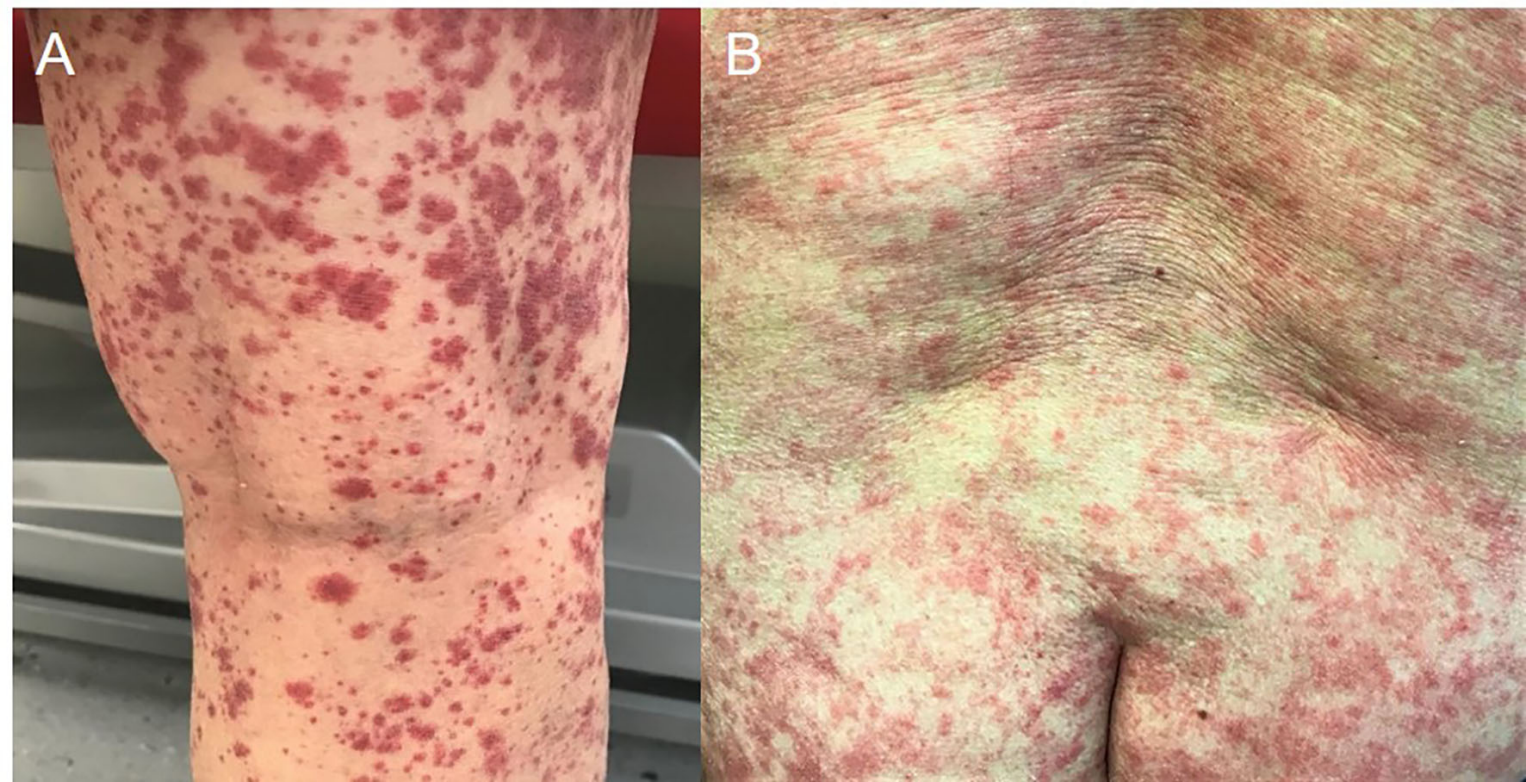
Skin examination: **(A)** lower legs **(B)** back.

**Figure 2:**
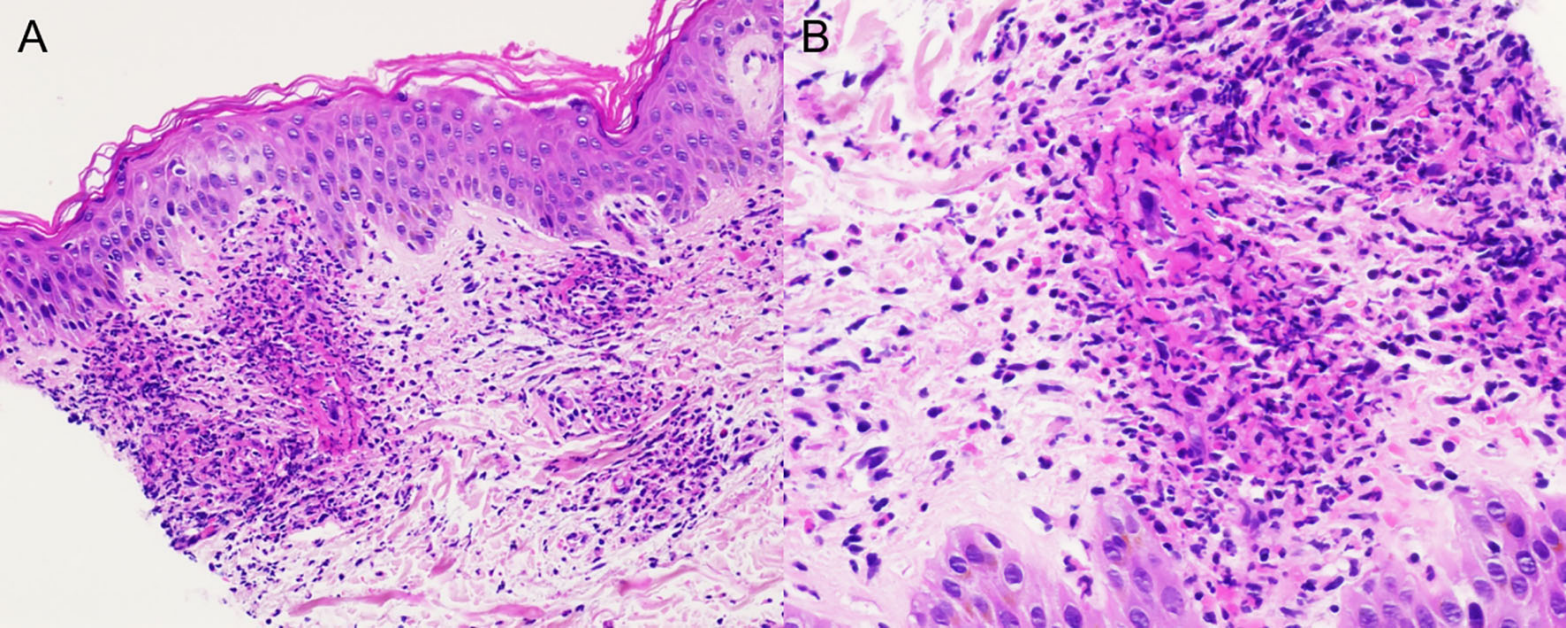
Skin sample showing intense neutrophilic infiltration of the blood vessels in the dermis with leukocytoclasis; **(A)** hematoxylin and eosin stain (HE) ×20, **(B)** HE × 40.

**Figure 3:**
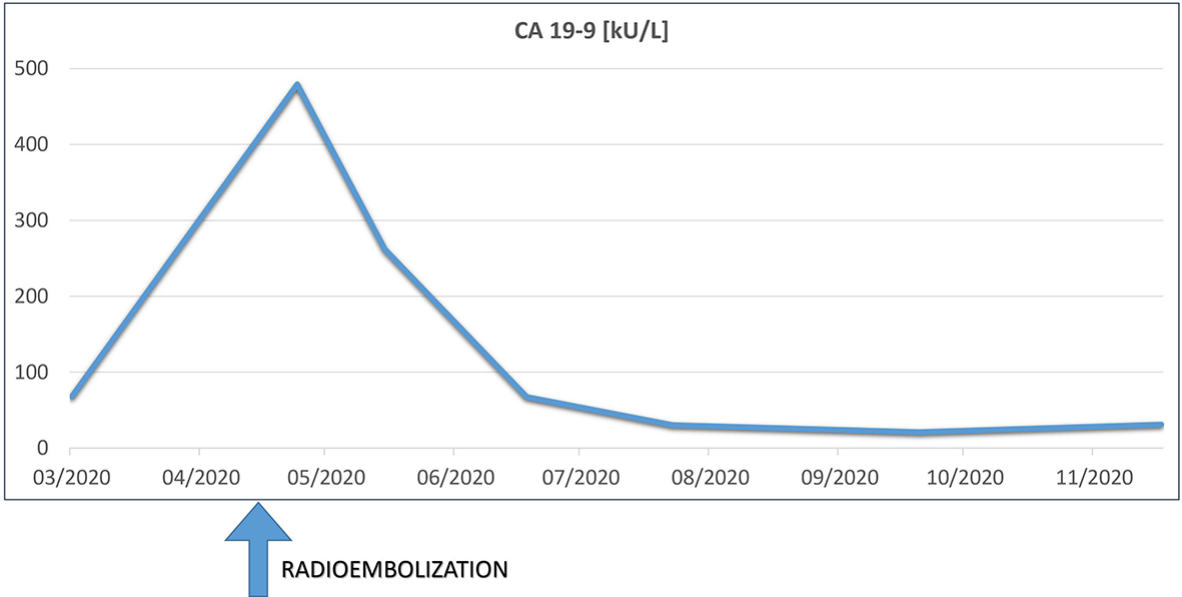
Serum levels of carbohydrate antigen 19-9 (CA 19-9) during treatment and follow-up.

Skin examination showed a purpuric maculopapular rash predominantly located on the lower legs (**Figure 1A**), thighs, buttocks, back (**Figure 1B**) and lower abdomen. No extracutaneous involvement was identified. Laboratory results were normal including hemoglobin, platelets, tryptase, IgE, liver enzymes and kidney function. Erythrocyte sedimentation rate was at 90 mm/hr and rheumatoid factor, antinuclear antibody, autoantibodies to neutrophilic cytoplasmic antigens were normal. CA 19-9 was elevated at 479 kU/l, while two months earlier, at baseline, it was 68 kU/l. A skin biopsy showed a positive immunofluorescence for IgM and C3 within vascular walls, without IgA involvement (thus excluding Henoch-Schönlein purpura) and pathologist concluded to a leukocytoclasic vasculitis (**Figure 2**). Screening for viral hepatitis had been negative. There was no recent change in his daily medication. He was started on colchicine and lesions completely resolved within 3 weeks. Tumor marker decreased by half at the time of vasculitis resolution, to 262 kU/l (**Figure 3**).

On follow-up 2 months post radioembolization, PET-CT showed new liver lesions and vertebral progression and the patient was treated with transarterial chemoembolization, spinal surgery and external beam radiotherapy, without recurrence of cutaneous vasculitis episodes. Unfortunately, the patient died a year after the diagnosis of cholangiocarcinoma.

## Discussion

Radioembolization or selective internal radiotherapy (SIRT) is a local treatment for hepatic tumors and metastases, using radiation-emitting isotopes like ^90^Y-labeled microspheres which are infused through the hepatic arteries. It offers the possibility of high-dose radiation administered directly to the tumor, sparing the surrounding tissues and thus leading to less liver toxicity compared to external beam radiation (3). Concerning intrahepatic cholangiocarcinoma, radioembolization can be used as a primary treatment, as an adjunct to chemotherapy or after chemotherapy failure. A recent phase 2 trial (MISPHEC) showed that patients receiving radioembolization combined with chemotherapy as first-line treatment in initially non-resectable intrahepatic cholangiocarcinomas reached a mOS of 22 months (95% CI, 14-52 months) and median progression-free survival (PFS) was 14 months (95% CI, 8-17 months). Response rates at 3 months was calculated at 39% according to RECIST criteria and 93% according to Choi Criteria and tumor downstaging for surgery was achieved for a large number of patients (4). A multicentric retrospective study showed that survival after radioembolization is influenced by disease extent and was worse in patients having received previous therapy (5). Most frequent toxicities of ^90^Y radioembolization include abdominal pain and elevated liver enzymes (6).

Concerning vasculitis, it refers to an inflammation of the vessel wall leading to its destruction and thickening, thus provoking lumen stenosis and ischemia of the surrounding tissues. Vasculitis can be idiopathic or triggered by drugs, infections, systemic diseases or localized pathologies. Cutaneous vasculitis manifests with urticaria, purpura, erythema, ulcerations, livedo reticularis and skin nodules. Diagnosis is confirmed by biopsy, but histologic findings should be interpreted in conjunction with history, clinical and laboratory findings as well as imaging (7).

Cutaneous vasculitis are primarily classified according to the size of the vessels involved (small and medium- vessel sized vasculitis) and secondarily distinguished by their underlying pathophysiological characteristics; for example: immune complex or autoantibodies involvement, as well as organs affected (8). In our case, the patient presented with leukocytoclastic vasculitis.

Leukocytoclastic vasculitis is a small vessel vasculitis that presents with palpable purpura localized mostly in the lower extremities. Symptoms are mainly restricted to the skin, although 30% of patients present with extracutaneous manifestations, including fever, weight loss, myalgia and arthralgia. Cutaneous symptoms include erythematous macules with palpable purpura often present bilaterally in the lower body, as well as hemorrhagic vesicles and bulla, pustules, nodules, ulcers, or livedo reticularis. The lesions can present at different stages of evolution (9). Half of leukocytoclastic vasculitis cases are idiopathic, while other ones are mainly secondary to drugs, infections, autoimmune diseases and malignancies. Cutaneous symptoms usually present one to three weeks after the provoking event, and the mechanism of pathogenesis involves deposition of immune complexes on vessel walls and complement activation. If the trigger is evident and in the absence of systemic symptoms, laboratory tests should include complete blood count, basic metabolic panel, liver function tests and urinalysis. Otherwise, further investigations are warranted, like viral hepatitis and HIV screening, serum protein electrophoresis, and workup for autoimmune diseases (10). Histopathological findings include neutrophilic infiltration of the vessel walls for new lesions (24 hours of onset) and lymphocytic infiltration for lesions present for longer than 24 hours (7, 9). Direct immunofluorescence should be performed with IgG, IgM, IgA and C3. Isolated IgA deposition suggests Henoch-Schönlein purpura (9). Idiopathic skin limited leukocytoclastic vasculitis usually resolve spontaneously. Treatment can include supportive measures such as leg elevation and compression stockings in order to prevent vessel dilation. Drugs that inhibit neutrophilic activity such as dapsone and colchicine may also be used. If a cause is recognized, it should be treated. Use of systemic corticosteroids is indicated in the presence of severe cutaneous symptoms such as hemorrhagic blisters or in case of severe systemic symptoms (11).

Paraneoplastic vasculitis account for less than 5% of vasculitis cases and are mainly reported with hematological malignancies. Solid tumors most often associated with vasculitis include lung, colon, prostate, breast and renal cancer, and the most frequent type of paraneoplastic vasculitis is also leukocytoclastic vasculitis (12). It has been hypothesized that paraneoplastic vasculitis is provoked either from reduced clearance of normal immune complexes, from formation of abnormal immune complexes that settle on the endothelium or formation of immunoglobulins against the tumor that also attack the vascular wall. Paraneoplastic vasculitis may precede, appear concurrently or after the cancer and can sometimes indicate a disease relapse. Treatment mainly consists of treating the underlying disease, though sometimes corticosteroids or other immunosuppressive treatments are necessary (13).

In the literature, we found 4 case reports of cholangiocarcinoma associated cases of vasculitis (**Table 1**). In one case, giant cell arteritis symptoms preceded cholangiocarcinoma diagnosis (12). In another case, cholangiocarcinoma presented with polyarteritis nodosa (15). Another patient presented pulmonary vasculitis before being diagnosed with cholangiocarcinoma (16). Lastly, a patient treated with cisplatin and gemcitabine combination for cholangiocarcinoma presented aortitis presumably associated with pegfilrastim (14).

**Table 1:** Cholangiocarcinoma and vasculitis.

Authors	Patient-age & sex	Vasculitis type	Timing	Cutaneous vasculitis symptoms	Extra-cutaneous vasculitis symptoms	Vasculitis therapy	Evolution
Saito et al. (14)	71 F	Pegfilgrastim-imduced aortitis	Vasculitis one week after 1^st^ line chemotherahy/1^st^ dose of pegfilgrastim	Absent	malaise, back and bilateral chest pain	Prednisone pegfilgrastrim eviction	Symptoms resolution
Solans-Laqué et al. (12)	83 F	Giant cell arteritis	Vasculitis 6 months before cancer (1)	Absent	Headache, scalp tenderness, neck/shoulder stiffness	Prednisone	Symptoms resolution and reactivation 5 months later while reducing prednisone
Hatzis et al. (15)	62 M	Polyarteritis	Vasculitis 8 months before cancer (2)	Dry gangrene middle finger, dark bulla at the lateral malleolus ulcerating	Legs numbness and pain, fever, chills	Methylprednisolone with cyclophosphamide	N/A (death 4 months after cholangiocarcinoma diagnosis)
Ong et al. (16)	62 F	Pulmonary vasculitis	Vasculitis 12 months before cancer	Absent	Pyrexia, malaise, progressive dyspnea	Prednisone	Respiratory symptoms resolution

F, female; M, male; N/A, not available.

(1) Giant cell arteritis leading to the discovery of a cholangiocarcinoma (paraneoplastic vasculitis).

(2) Polyarteritis nodosa symptoms leading to the discovery of a cholangiocarcinoma (paraneoplastic vasculitis).

We found no literature concerning vasculitis induced by radioembolization or radiofrequency ablation. Nevertheless, we found some data concerning chemoembolization local vasculitis induced in the infused vessel (17, 18). We found 3 articles in the literature describing vasculitis as a clinically significant complication of chemoembolization (19–21) (**Table 2**) but only in one case were details of this complication described; a 45 year old patient who presented paraparesis attributed to anterior spinal artery vasculitis caused by chemotherapy (21).

**Table 2:** Chemoembolization & Vasculitis.

Authors	Patient-age & sex	Vasculitis type	Setting	Timing	Cutaneous vasculitis symptoms	Extra-cutaneous vasculitis symptoms	Vasculitis therapy	Therapy efficacy
Tufail et al. (21)	45 M	Toxic vasculitis	CHC treated by TACE; localized vasculitis of the branches of the anterior spinal artery, caused by the chemotherapy, leading to paraparesis (spinal cord injury)	8 hrs after TACE	Absent	Bilateral lower extremity weakness	High-dose steroids	Resolution over ~1 month
Giannelli et al. (22)	75 M	Leukocytoclastic vasculitis	Hepatocellular carcinoma treated by TACE	10 days after TACE	Palpable purpuric lesions on abdomen, thighs and calves	Absent	Supportive treatment	N/A
Morino et al. (19)	N/A, 5 patients	N/A	Preoperative chemoembolization for hepatocellular carcinoma	After TACE	N/A	N/A	N/A	N/A
Bismuth et al. (20)	N/A, 40 patients (14% of 291 patients)	Mechanical or toxic	Primary treatment of hepatocellular carcinoma by arterial chemoembolization	After TACE	N/A	N/A	N/A	N/A

F, female; M, male; N/A, not available.

In regard of the chronology of events in our case, it seems that the vasculitis was triggered by the ^90^Y radioembolization. This could suggest a type B adverse drug reaction (ADR) defined as unpredictable, dosage independent, with low morbidity, related not to therapeutic effectiveness but rather to the host.

We excluded the contrast agent as a vasculitis trigger because there was no skin modification after the macroaggregated albumin (^99^mTc-MAA) test which used the same contrast media.

Moreover, we excluded a paraneoplastic vasculitis as the Curth criteria were not fulfilled, like the absence of temporal relationship (the vasculitis was not present before the cancer diagnosis and did not reoccur at progression).

We also do not consider a medication etiology since no drugs were introduced or modified in this setting. There was also no infection or proof of concurrent autoimmune disease.

## Conclusion

We presented the case of an 88 year-old patient who presented with cutaneous vasculitis shortly after ^90^Y radioembolization. In the absence of other evident triggers, we suggest that this leukocytoclastic vasculitis was caused by the ^90^Y radioembolization and, to our knowledge, is the first case reported in the literature, as well as the favorable evolution with colchicine. As a rare complication, a multidisciplinary approach is indicated between oncologists, interventional/nuclear radiologists and more data are needed to improve our care.

This paper has attempted to characterize the cutaneous vasculitic reaction seen with the use of ^90^Y in the hope of aiding its early recognition and treatment.

## Data Availability Statement

The original contributions presented in the study are included in the article. Further inquiries can be directed to the corresponding author.

## Ethics Statement

Written informed consent was obtained from the individual(s) for the publication of any potentially identifiable images or data included in this article.

## Author Contributions

AS, JJ, PS, and ADig drafted the manuscript. All authors provided critical feedback and approved the version to be published. FF provided the pathology images for **Figure 2**.

## Conflict of Interest

The authors declare that the research was conducted in the absence of any commercial or financial relationships that could be construed as a potential conflict of interest.

## Publisher’s Note

All claims expressed in this article are solely those of the authors and do not necessarily represent those of their affiliated organizations, or those of the publisher, the editors and the reviewers. Any product that may be evaluated in this article, or claim that may be made by its manufacturer, is not guaranteed or endorsed by the publisher.
